# Characterization of the complete mitochondrial genome of *Anomala antiqua* (Coleoptera: scarabaeidae) and its phylogenetic implications

**DOI:** 10.1080/23802359.2026.2642518

**Published:** 2026-03-11

**Authors:** Shuang Cao, Xiaojing Zhou, Zhigang Yin, Na Ma, Peiyu Chen

**Affiliations:** a Institute of Chinese Medicinal Materials, Nanyang Academy of Sciences, Nanyang, China; b College of Water Resources and Modern Agriculture, Nanyang Normal University, Nanyang, China

**Keywords:** Rutelinae, Anomalini, mitogenome, phylogenetic analysis

## Abstract

The complete mitochondrial DNA (mtDNA) of *Anomala antiqua* (Gyllenhal, 1817) (Coleoptera: Scarabaeidae) was first described by next-generation sequencing in this study. The length of mitogenome is 16,430 bp with AT content of 77.3%, which contained 37 genes, including 13 protein-coding genes (PCGs), 22 transfer RNA genes (tRNAs) and two ribosomal RNA genes (rRNAs). Gene order is conserved and identical to most other previously sequenced Rutelinae. Phylogenetic analysis based on the amino acid sequences of 13 PCGs reveals that *A. antiqua* forms a basal branch of the subfamily Rutelinae and the genus *Anomala* is paraphyletic. The complete mtDNA of *A. antiqua* will be an important genomic resource for molecular identification and systematic classification of the genus *Anomala*, offering valuable insights into the evolutionary history and taxonomic status of its species.

## Introduction

The subfamily Rutelinae (Coleoptera: Scarabaeidae) is globally distributed and comprises seven tribes, with over 235 genera and more than 4,200 species (Bouchard et al. 2011). Among these, the tribe Anomalini is the most speciose, containing approximately 54 genera and 2,000 species (Lu et al. 2023). It is primarily distributed across the Oriental, Palearctic, and Afrotropical regions (Jameson et al. 2007; Zhao et al. 2024). This tribe includes the genus with the highest species richness within the Rutelinae subfamily: *Anomala* Samouelle, 1819 which has over 1,000 recorded species (Wang et al. 2024). *Anomala* beetles are major agricultural and forestry pests, with adults feeding on plant leaves and flowers, and the soil-dwelling larvae consuming plant roots. This causes severe damage to crops and forests, resulting in substantial economic losses annually (Lu et al. 2023). However, the remarkable similarity in external morphology among these insects makes it extremely difficult to distinguish closely related species, leading to challenges in both precise control and classification (Lu et al. 2023). The mitochondrial genome, due to its small molecular size (typically 15-20 kb), stable structure, moderate evolutionary rate, and maternal inheritance characteristics, is utilized in species identification, population genetics, and phylogenetic research (Cameron 2014). At present, mitochondrial genomes are available only for a few species within Rutelinae (Qu et al. 2023).

In this study, the mitochondrial genome of *Anomala antiqua* (Gyllenhal, 1817) was sequenced by high-throughput sequencing technology, assembled, and analyzed. A phylogenetic tree was constructed and analyzed based on the known mitochondrial genome data of the Rutelinae. This study will provide crucial data support for the molecular identification and phylogenetic research of the genus *Anomala*.

## Materials and methods

**Figure 1.**
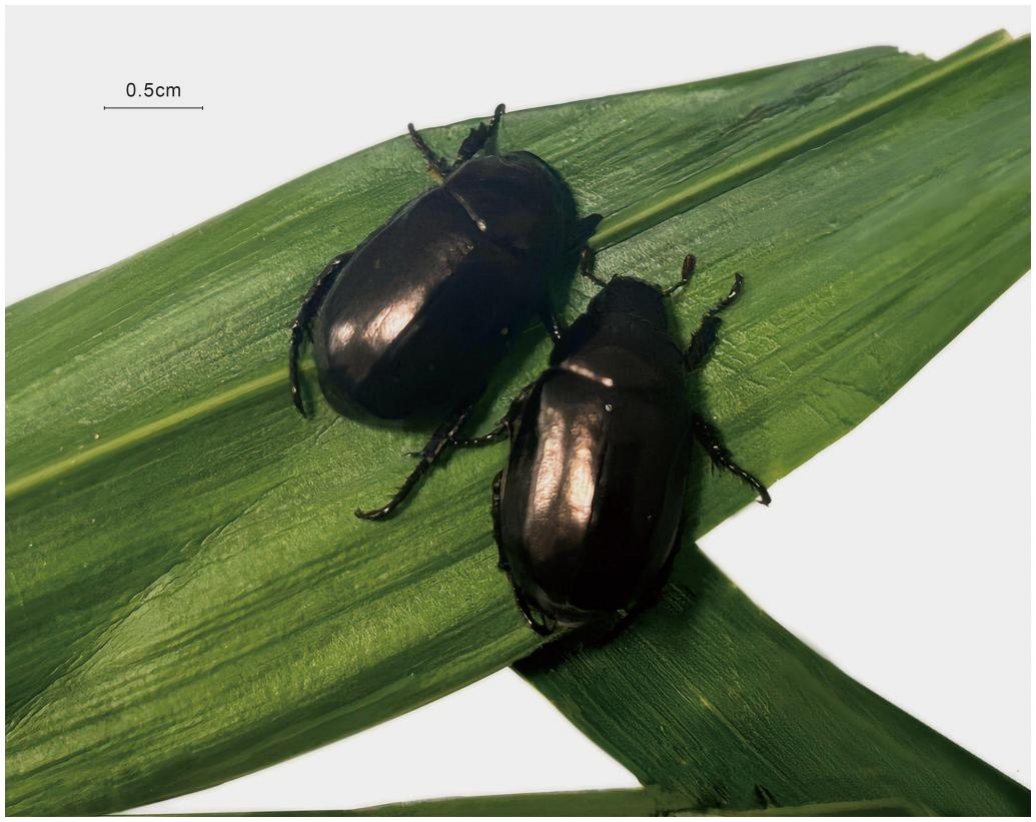
Photograph of *anomala antiqua* adult habitus. Specimens for this study were collected in Nanyang by Peiyu Chen. The photo was taken in Nanyang by Peiyu Chen.

Samples of *A. antiqua* were collected on 19 June 2025 in Nanyang City (32.907902°N, 112.428497°E), Henan Province, China, and twenty individuals were used for subsequent experiments. Adult specimens were identified morphologically and photographed using digital camera (Canon EOS R50) (Figure 1). The samples were fixed in 99.9% ethanol and stored at the Nanyang Academy of Sciences, under the voucher number Aa 0001 (contact Peiyu Chen, chenpeiyu1984@163.com).

Total genomic DNA was extracted from the thoracic muscle tissue of the samples using the TIANamp Genomic DNA Kit (Tiangen Biotech, Beijing, China). After fragmentation, the paired-end sequencing library was constructed using the VAHTS Universal Plus DNA Library Prep Kit for Illumina (Vazyme, Nanjing, China; Cat. No. ND617-02), following the manufacturer’s instructions. Sequencing was performed on the Illumina NovaSeq 6000 platform (Kai Tai Mingjing Gene Technology Co., Ltd. Beijing, China) with a 150 bp paired-end strategy, and generated approximately 31.39 Gb raw data including 209,291,800 raw reads. Quality control and filtering of the raw paired-end sequencing data were conducted using fastp 0.23.2 (Chen 2023), including adapter removal and elimination of low-quality reads. The mitochondrial genome was assembled using GetOrganelle v1.7.7.1 software aligned with homologous sequences in the GenBank database to verify the accuracy of the sequencing results (Jin et al. 2020). Annotation of the mitochondrial genome was performed using MitoZ 3.6 (Meng et al. 2019). The complete mitochondrial genome sequence was submitted to GenBank under the accession number (PX659699).

For phylogenetic analysis, the amino acid sequences of 13 protein-coding genes (PCGs) were extracted from the complete mitogenomes of 11 previously reported Rutelinae subfamily from GenBank and the genome sequenced here from the genus *Anomala* as the ingroup for analysis, with the *Xylotrupes socrates tonkinensis* Minck, 1920 as the outgroup. Each PCG sequence was separately aligned by MAFFT v7 (Katoh and Standley 2013), trimmed with Gblocks v0.91b (Talavera and Castresana 2007) using default parameters and concatenated to create a single dataset. Best-fitting amino acid substitution models were determined for each PCG using PartitionFinder2 v2.1.1 (Lanfear et al. 2017) under the Bayesian Information Criterion. Maximum-likelihood (ML) and Bayesian inference (BI) trees were then generated.

The ML analysis was conducted using by RAxML-NG v8 (Edler et al. 2021) with 1,000 bootstrap replicates under the selected partitioning scheme and models. The BI analysis was performed using MrBayes v3.2.7a (Ronquist et al. 2012) with two independent runs of 2 million generations each, sampling every 100 generations. The first 25% of trees were discarded as burn-in after confirming convergence (average standard deviation of split frequencies < 0.01). Final phylogenetic trees were visualized and beautified using iTOL v6 (Letunic and Bork 2024).

## Results

### Mitochondrial genome structure of *Anomala antiqua*

**Figure 2.**
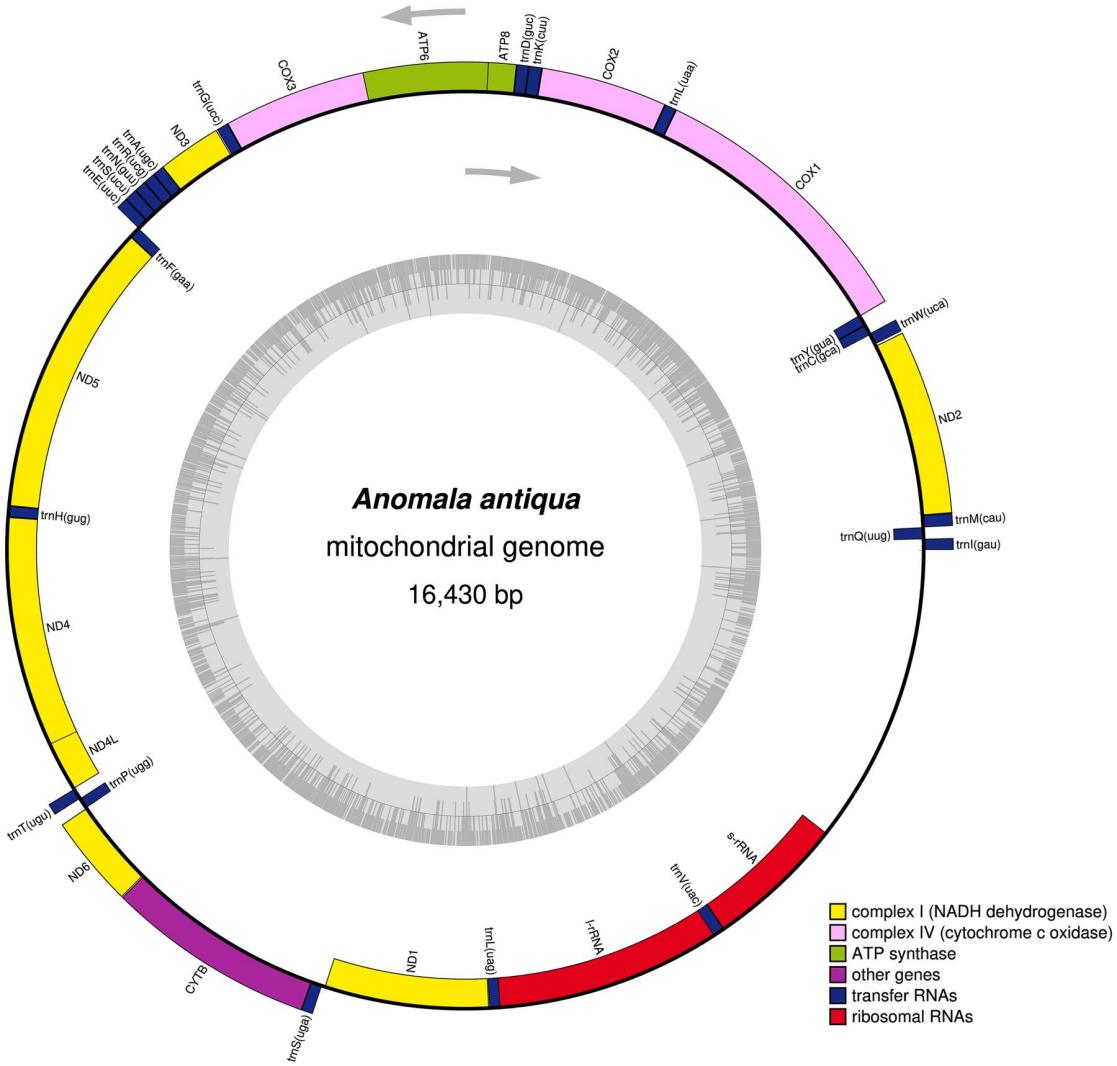
*Anomala antiqua* mitochondrial genome map. Genes encoded on the plus strand are indicated in the inner circle, genes encoded in the minus strand are indicated in the outer circle.

The complete mitochondrial genome of *A. antiqua* is 16,430 bp in length and encodes a typical set of 37 genes, including 13 PCGs, 22 transfer RNA (tRNAs) and 2 ribosomal genes (rRNAs) (Figure 2). The mitochondrial DNA of *A. antiqua* was successfully assembled with high coverage (Figure S1). The heavy strand (H-strand) contains 23 genes, including 9 PCGs (nd3, cox3, atp6, atp8, cox2, cox1, nd2, cytb, nd6) and 14 tRNAs. The light strand (L-strand) contains the remaining 14 genes: 4 PCGs (nd5, nd4, nd4l, nd1), 8 tRNAs and 2 rRNAs. The mitogenome of *A. antiqua* exhibits a strong AT-bias, with nucleotide composition of 39.8% A, 37.5% T, 8.9% C and 13.7% G. The total length of 13 PCGs was 11,179 bp, ranging from nd5 (1,716 bp) to atp8 (156 bp). Consistent with other Rutelinae mitogenomes, no gene rearrangement or deletion was observed.

### Phylogenetic analysis

**Figure 3.**
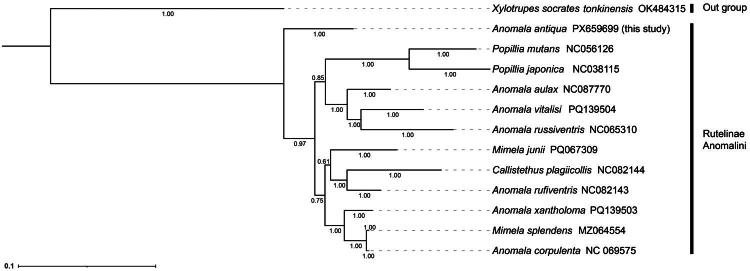
Bayesian inference (BI) phylogenetic tree based on amino acid sequences of 13 PCGs. The tree was reconstructed using amino acid sequences translated from the 13 protein-coding genes of complete mitochondrial genomes. *Xylotrupes socrates tonkinensis* was chosen as the outgroup. The numbers on branches indicate posterior probabilities from Bayesian inference (decimal values). The following sequences were used: *Popillia japonica* NC038115 (Yang et al. 2018), *Popillia mutans* NC056126 (Song and Zhang 2018), *Anomala antiqua* PX659699 (this study), *Anomala corpulenta* NC069575 (Qu et al. 2023), *Anomala rufiventris* NC082143 (Long et al. 2024), *Anomala russiventris* NC065310 (Li et al. 2022), *Mimela junii* PQ067309 (Nardi et al. 2024), *Mimela splendens* MZ064554 (unpublished), *Callistethus plagiicollis* NC082144 (Long et al. 2024), *Anomala vitalisi* PQ139504 (unpublished), *Anomala xantholoma* PQ139503 (unpublished), *Anomala aulax* NC087770 (unpublished), and *Xylotrupes socrates tonkinensis* OK484315 (unpublished).

Phylogenetic analysis showed that the tree topologies generated by the two methods were consistent, except for the position of *Mimela junii* (Duftschmid, 1805) (Figure 3 and Figure S2). In both trees, *A. antiqua* was located at the basal clade of Anomalini, forming a sister group to all remaining species. The monophyly of the genus *Popillia* Serville, 1825 was highly supported (PP = 1). Three species of the genus *Anomala*, *A. aulax* (Wiedemann, 1823), *A. vitalisi* Ohaus, 1914, and *A. russiventris* Fairmaire, 1893 formed a sister group relationship with *Popillia*, while the genera *Anomala* and *Mimela* Kirby, 1823, were considered as paraphyletic (Figure 3). The other six species, belonging to the genera *Mimela*, *Anomala*, and *Callistethus* Blanchard, 1851, clustered into a single clade. However, their relationship remains unresolved as they were not distinctly separated by genus. This indicates a complex or ambiguous phylogenetic relationship among these taxa.

## Discussion and conclusion

This study presents the first complete mitochondrial genomes characterization of *A. antiqua* from China. The mitochondrial genome length of *A. antiqua* is 16,430 bp, which is similar to that of other three reported scarab beetles *A. russiventris* (15,601 bp) (Li et al. 2022), *A. corpulenta* Motschulsky, 1854 (16,773 bp) (Qu et al. 2023), and *A. rufiventris* Kollar & Redtenbacher, 1844 (17,240 bp) (Long et al. 2024). It comprises 37 mitochondrial genes, exhibiting a gene arrangement consistent with that of other Rutelinae mitochondrial genomes.

The tribe Anomalini comprises a substantial number of species, accounting for over half of all known Rutelinae species. However, its phylogenetic position and the relationships among its constituent genera and species remain poorly understood (Lu et al. 2023). Previous studies have suggested that the genus *Anomala* shares a closer phylogenetic relationship with the genus *Mimela* than with *Popillia* (Qu et al. 2023). Our phylogenetic analysis based on 13 PCGs sequences from 12 species within this subtribe revealed that the intergeneric relationships within this tribe remain unresolved as the genus *Anomala* is as paraphyletic, consistent with other recent phylogenetic studies (Song and Zhang 2018; Li et al. 2022; Qu et al. 2023; Long et al. 2024; Nardi et al. 2024).

Based on our phylogenetic reconstruction, *A. antiqua* occupied a basalmost position within tribe Anomalini, highlighting its pivotal role in the phylogeny of the group. The complete mtDNA of *A. antiqua* reported in this study and phylogenetic results will play an important role in understanding the complex species definition evolution and systematic relationships of genus *Anomala*. However, given the high species diversity within the Rutelinae subfamily, future studies should incorporate a broader range of taxa and integrate morphological characteristics with multiple genetic markers to more comprehensively and systematically elucidate their phylogenetic relationships and evolutionary patterns.

## Supplementary Material

Supplemental Material

Supplemental Material

## Data Availability

The mitochondrial genome data are available with the accession number of PX659699 in the GenBank of NCBI (https://www.ncbi.nlm.nih.gov/). The associated BioProject, SRA, and Bio-Sample numbers are PRJNA1354598, SRR35900079, and SAMN52949802, respectively.
