# Supplementary figures and images for: Characterization of the complete mitochondrial genome of Anomala antiqua (Coleoptera: scarabaeidae) and its phylogenetic implications
Source: Mitochondrial DNA B Resour. 2026 Mar 11;11(4):527–30. doi: 10.1080/23802359.2026.2642518 (PMC12981256; doi:10.1080/23802359.2026.2642518)

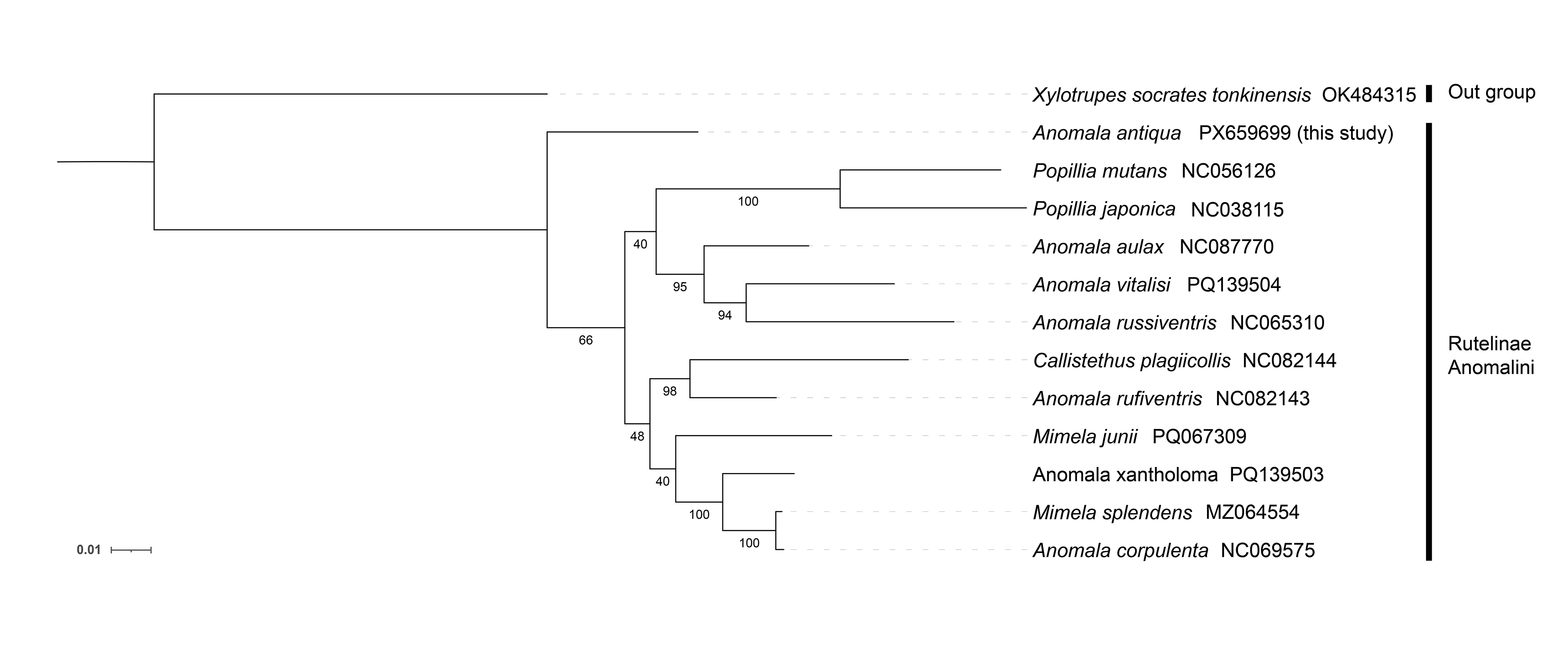

Supplement: Supplemental Material [file TMDN_A_2642518_SM3294.jpg]

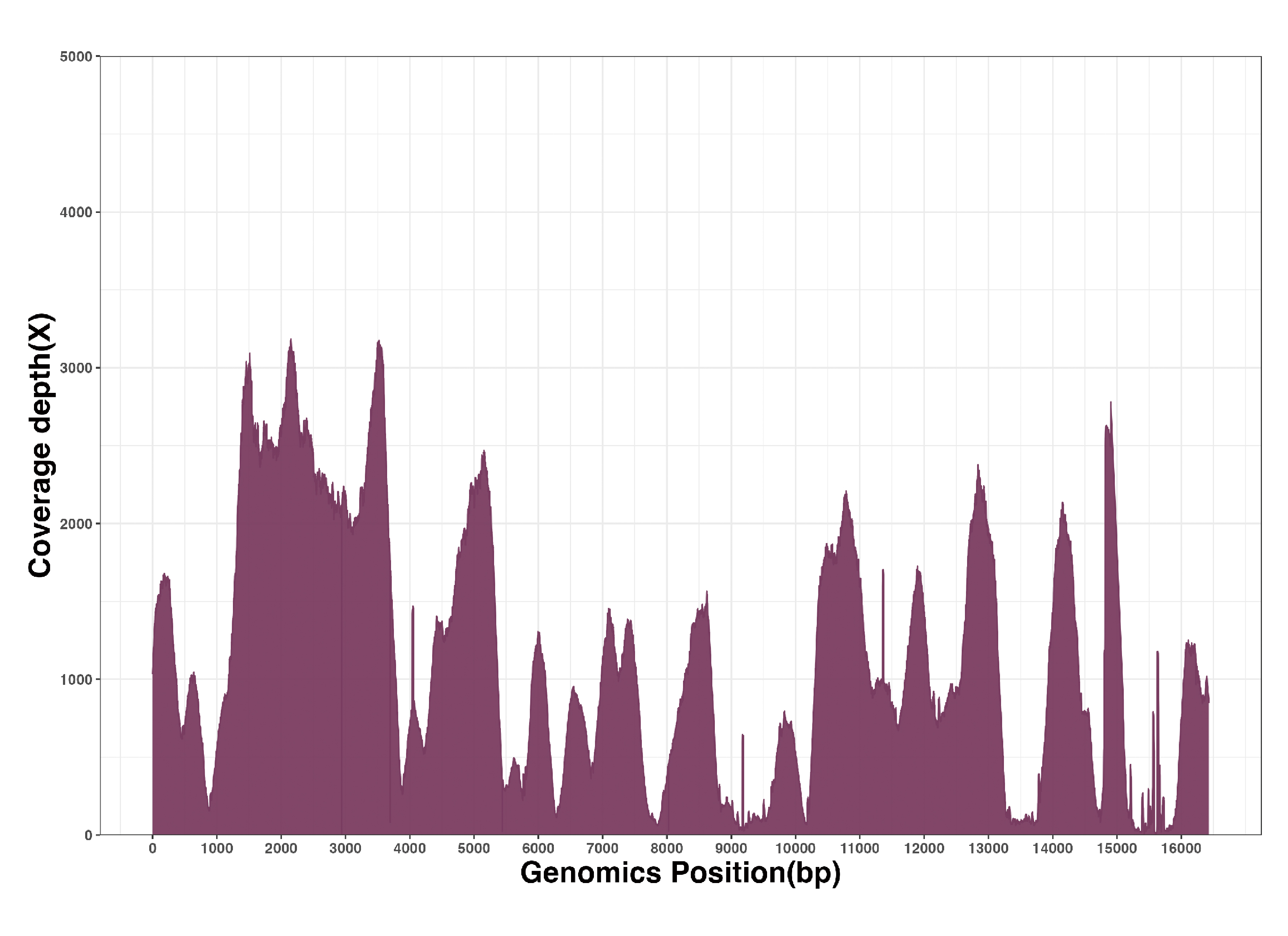

Supplement: Supplemental Material [file TMDN_A_2642518_SM3293.jpg]
